# Problems in Classifying Mild Cognitive Impairment (MCI): One or Multiple Syndromes?

**DOI:** 10.3390/brainsci7090111

**Published:** 2017-09-01

**Authors:** María del Carmen Díaz-Mardomingo, Sara García-Herranz, Raquel Rodríguez-Fernández, César Venero, Herminia Peraita

**Affiliations:** 1 Department of Basic Psychology I, National University of Distance Education, Juan del Rosal 10, 28040 Madrid, Spain; sgarcia@bec.uned.es (S.G.-H.); peraitaherminia@gmail.com (H.P.); 2 Department of Behavioural Sciences Methodology, National University of Distance Education, Juan del Rosal 10, 28040 Madrid, Spain; rrodriguez@psi.uned.es; 3 Department of Psychobiology, National University of Distance Education, Juan del Rosal 10, 28040 Madrid, Spain; cvenero@psi.uned.es

**Keywords:** classification methods, mild cognitive impairment, neuropsychological assessment, social factors, loneliness, subtypes

## Abstract

As the conceptual, methodological, and technological advances applied to dementias have evolved the construct of mild cognitive impairment (MCI), one problem encountered has been its classification into subtypes. Here, we aim to revise the concept of MCI and its subtypes, addressing the problems of classification not only from the psychometric point of view or by using alternative methods, such as latent class analysis, but also considering the absence of normative data. In addition to the well-known influence of certain factors on cognitive function, such as educational level and cultural traits, recent studies highlight the relevance of other factors that may significantly affect the genesis and evolution of MCI: subjective memory complaints, loneliness, social isolation, etc. The present work will contemplate the most relevant attempts to clarify the issue of MCI categorization and classification, combining our own data with that from recent studies which suggest the role of relevant psychosocial factors in MCI.

## 1. Introduction

Mild cognitive impairment (MCI) was initially described as a single syndrome in which the individual displays: (a) subjective memory complaints, confirmed by a reliable informant; (b) objective deficits in episodic memory tests with no impairment in daily living; and of course, (c) no dementia [1]. Since then, the concept of MCI has gradually diversified into distinct subtypes, with different classifications arising according to its cognitive characteristics [1,2], clinical presentation [3], and probable etiology [4]. In addition, longitudinal studies have revealed that not all types of MCI evolve to dementia, as was initially thought, an issue that has yet to be fully resolved.

In terms of cognitive characteristics, MCI was initially defined solely on the basis of an assessment of episodic memory [2], indicating that MCI was of an amnestic nature (aMCI). That is, cognitive problems were only revealed as a function of episodic memory (as long as the score obtained in these tests was 1.5 Standard Deviation—SD—below the mean) without taking into consideration alterations to other cognitive functions. This very restrictive initial notion of MCI was extended when three different MCI subtypes were proposed (amnestic, multidomain, and single domain MCI without memory deficit) [5,6], which were later reclassified or subdivided into four subtypes depending on the number of domains affected: single domain aMCI, multidomain aMCI, single domain non-amnestic MCI (naMCI), and multidomain naMCI [7]. In 2004, it was proposed that MCI could be classified into three subtypes [3]: aMCI, multiple domain, and single non-memory domain. Moreover, it was considered that MCI should be subdivided according to its distinct clinical presentation and/or etiology: MCI due to cerebrovascular disease, MCI due to Parkinsonism disease (PD), MCI with depressive symptomatology, or MCI with psychological and behavioral symptomatology. It was explicitly qualified that although neurodegeneration may be the underlying etiology behind MCI, the deterioration of memory could evolve as a consequence of other clinical conditions.

Currently, the majority of researchers employ the four subtypes of MCI described [7,8,9,10,11,12]. However, some researchers also take into account the amnestic alteration and its different modalities, extending the classification of MCI according to the deterioration of other cognitive functions and capacities (see Table 1): executive, language, and processing speed [8,13,14,15,16,17]. In addition to the aforementioned subtypes, undifferentiated MCI characterizes individuals with subjective cognitive complaints but with no cognitive impairment evident in cognitive tests [18]. Specifically, this fourth classification was defined because, although it applies to individuals with cognitive impairment, its characteristics do not match any of the three former types. Indeed, this group is that most affected when taking a variation of 1.5-SD as the threshold instead of 1-SD. Two possible hypotheses have been proposed to interpret this type of undifferentiated MCI: that it may subsequently progress to Alzheimer’s disease (AD) or conversely, that it might be a subtype that evolves to normality. Irrespective of the subtypes of MCI considered, it is clearly necessary to examine the subcomponents or facets of executive function that are involved in greater depth (those affected in at least two of these subtypes), information that is essential to characterize the different subtypes and to allocate individuals to them [19].

Despite the increase to four subtypes of MCI proposed over the years, there are still authors who prefer to use a classification that only considers two major groups in function of the status of episodic memory, aMCI, and naMCI [20,21,22,23,24]. Similarly, in the COSMIC Study (Cohort Studies on Memory in an International Consortium) that was the result of 11 international cohort studies [25], and despite recognizing the enormous potential of the different MCI subtypes for both clinical diagnosis and prognosis, objective MCI was classified as these two classic types.

Surprisingly, in a European Union (EU) report on MCI in longitudinal cohorts, there is no reference to MCI subtypes [26]. Biological risk factors do appear and are considered distinct from the cognitive, behavioral, or functional factors. In addition, this report grants importance to the need to assess the cognitive aspects that are currently being poorly evaluated in greater depth, such as motor and perceptual aspects or processing speed, which could represent early indicators of cognitive decline, as well as the difference between modifiable risk factors and protective factors. A review of the concept and vocabulary in AD only attended to the definition of aMCI [27], although it established—and this is interesting for our purposes—that since the conceptualization of prodromal AD has changed, MCI must focus on something much less specific.

Thus, we note a tendency in the current scientific literature to acknowledge the need to define and characterize at least one new type of MCI, one that is less clearly differentiated and with no apparent objective etiology, and that may be due to the interaction of many variables or factors but that does not necessarily lead to dementia. Nevertheless, this must be addressed while explicitly maintaining the objective classic typology of the four (or two) original sub-types of MCI as they can be linked to biomarkers [28]. However, this does not seem to be consistent, at least not fully, with the minor cognitive disorder named ‘Mild NeuroCognitive Disorder’ (mNCD) that was included by the American Psychiatric Association (APA) in the Diagnostic and Statistical Manual of Mental Disorders (DSM-5) [29]. Yet while this disorder is somehow related to MCI, the diagnostic agreement between both conditions seems to be limited [30].

**Figure 1:**
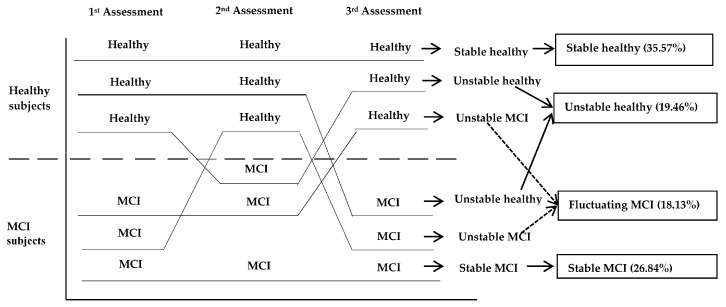
Heterogeneity of the cognitive trajectories in cognitively healthy older adults and MCI subjects in a year-year follow-up study. Doctoral Thesis, García-Herranz [39].

Etiology is another classification criterion that must be taken into account and in fact, it is considered a determinant factor in the 2015 updated DSM-5 [31]. Indeed, the classification of MCI as MCI-AD, vascular MCI, or MCI-Lewy body dementia has been proposed [4]. Similarly, the importance of reclassifying individuals according to the degree of diagnostic certainty has also been raised: possible MCI, when co-morbidities exist that may explain or contribute towards cognitive deficits (e.g., cardiovascular diseases, a history of cranioencephalic trauma, infectious diseases, psychiatric disorders, etc.), or probable MCI, when no such co-morbidities may influence diagnosis [32,33]. According to the evolutionary course of MCI, and in view of different longitudinal studies [11,34,35,36,37,38], it has been verified that MCI can follow different evolutionary trajectories: (a) stable, with cognitive impairment maintained across different domains over time without conversion to dementia or without cognitive improvement; (b) unstable, with fluctuating changes over time between cognitive normality and MCI; (c) remitting, with the recovery of normal cognitive function; and (d) progressive, those that progress to dementia (see Figure 1).

Data from longitudinal research at the Karolinska Institute, the Kungsholmen Project, suggests the existence of a type of MCI called Cognitive Impairment No Dementia (CIND), with three degrees of severity: mild, moderate, and severe (although only based on the MMEE) [40]. A quarter of the individuals diagnosed with CIND reverted to normality within three years, or their cognitive function improved, and only one third of these individuals evolved to dementia. On the basis of this data, it was indicated that MCI is much more heterogeneous than had been described to date, although the existence of subtypes was not addressed. In addition, different aspects of the individual’s personality—depressive states, anxiety, cultural level, loneliness—were recognized as factors that may determine the undifferentiated type of MCI.

MCI has also been restricted to an underlying neurodegenerative pathology that can be associated with biomarkers [12]. However, the door was left open—albeit implicitly—to a new characterization and the hope of discovering specific biomarkers in the future for this undifferentiated and increasingly accepted type of MCI.

## 2. Importance of Neuropsychological Assessment in Determining MCI Subtypes

Despite the heterogeneity of MCI, there is a consensus among clinicians and researchers to consider cognitive assessment as an essential element in the diagnosis of MCI and its classification into subtypes [12,38,39,40,41]. The diagnosis of MCI is not only based on cognitive assessment, often performed through a short screening and with the limitations that this implies [42] but also takes into account the individual’s medical history, and their physical and functional status, as well as complementary medical tests [43]. Accordingly, comprehensive cognitive assessments, not only through screening tests but also through well-designed assessment batteries, should detect deficits in various cognitive processes and allow different subtypes of MCI to be established [44,45,46].

Each type of study—pidemiological, normative, clinical, etc.—influence the weight that is given to the neuropsychological assessment. In studies aimed at an early detection of MCI in cohorts of older adults, apparently cognitively healthy, diagnostic criteria center on cognitive and neuropsychological criteria, and accordingly, the cognitive assessment is crucial. This assessment involves applying a protocol with a varied theoretical-conceptual structure and a number of tests that evaluate distinct processes, functions, areas, or cognitive domains. The lack of consensus on which domains should be assessed and which tests should be used is the reason why a standardized internationally agreed protocol still does not exist [12,46,47]. Furthermore, there is no consensus on the cut-off points or thresholds to differentiate normal from pathological cognitive performance, contributing to the wide disparity of outcomes in this research field [10,48,49,50,51].

In studies that have based MCI diagnosis only on the performance of individuals in cognitive screening, MCI has been diagnosed or detected in individuals yielding an unexpected performance due to poor education, sensory deficits, etc., resulting in false positives [10,38,52]. In 2011, basic clinical criteria for MCI were proposed by the Spanish Society of Neurology (SEN) [53] and by the National Institute on Aging-Alzheimer’s Association workgroup (NIA-AA) [20], whereby cognitive impairment must be detected across one or more domains. In this sense, assessment should include tests that evaluate performance in cognitive domains other than memory, such as attention/executive function, language, and visuoperceptual/visuoconstructional functions [54]. In addition, it is rare to find a definition of the number of tests or subtests explicitly required to define deterioration in a cognitive domain. Poor performance in a cognitive domain can be considered when the person fails a single test, or execution in that domain may be assumed to be altered if there is deficient performance in two or more tests that evaluate that same cognitive domain. The diversity of tests used to evaluate such domains also poses a new problem as they are not all designed on the basis of the same theoretical proposition. Hence, the extension, complexity, and number of subtests included may differ. This factor greatly complicates any comparison of results between different studies, even when the tests used to evaluate the different domains are explicitly indicated.

Although the assessment of verbal episodic memory is essential as it may be a central marker for AD [55,56,57], and should therefore be included in studies employed to detect MCI, its assessment is not always performed in the same way or with the same rigor. This variability in verbal episodic memory assessment is related, in part, to the large number of tests available, which differ in the number of items and the availability of cues for memory retrieval, as well as the degree of involvement of executive and/or linguistic components. In addition to episodic memory, it is also necessary to assess executive functioning, attentional ability, and processing speed in order to characterize the different MCI subtypes [14,49,58,59]. Furthermore, the importance of assessing language ability should not be overlooked, as verbal fluency tests (namely semantic fluency), as well as memory and executive functioning tests, are significant predictors of future cognitive decline [60,61]. Regarding visuospatial ability, research on visuoconstructive deficits is scarce even though this domain deteriorates early in people with MCI who convert to AD [62,63].

It is therefore clear that the number and variety of neuropsychological instruments used to assess MCI, and its theoretical conception, can help to determine the subtypes of this clinical entity. Hence, it would be reasonable and helpful to be able to achieve guidelines that would clearly state which cognitive domains and how many tests in each domain should be used to establish MCI subtypes [12]. The criteria could vary in terms of the number of tests (one or two) which the individual must fail in a given cognitive domain (with a prior determination of the domains) and on the level of performance or the cut-off point that defines deterioration in any test (1.0, 1.5 or 2 SD) [64]. There are two classic operative criteria for MCI diagnosis based on the combination of both these criteria: the SD and the number of tests to be considered. Firstly, the Petersen/Winblad conventional criteria adopt a threshold of 1.5 SD below the mean and the failure of a single test per cognitive domain. Alternatively, the Jak/Bondi criteria center on 1.0 SD below the mean and incorrect performance in two or more tests per cognitive domain. Several studies have compared these criteria, such as a study to demonstrate the effects of different operational definitions of MCI on prevalence estimates in older adults [65]. MCI prevalence in the sample was highly dependent on these variables, and varied from 11% to 92% of the sample. The use of a less severe impairment cut-off (1.0 SD) and the impairment in only one test yielded higher rates of MCI. The Petersen/Winblad and Jak/Bondi criteria have also been compared directly, whereby the Petersen/Winblad criteria classified 34% of participants as having MCI, while the Jak/Bondi criteria classified only 24% as having MCI [10]. Therefore, it is evident that the variability in the operative/diagnostic criteria of classification will affect the estimated prevalence of MCI and the subtypes derived from it, with important psycho-social, clinical, and epidemiological consequences, among others. Thus, the classification of MCI into subtypes is conditioned by multiple factors, including the amplitude of the neuropsychological assessment, the selection of tests, and the cut-off points chosen.

There is little consensus on how the factors described above influence early MCI detection and different approaches have tried to address this problem. We specifically started to design a battery of tests in 2005, in line with other longitudinal studies that had a similar objective, in which the selection of tests or subtests was based on those that were considered to be the most sensitive predictors for the diagnosis of MCI at that time. Therefore, we selected the Verbal Learning Test Spain Complutense (Test de Aprendizaje Verbal España Complutense—TAVEC) [66], a semantic and a phonemic verbal fluency test [67], the Trail Making Test A and B [68], certain praxis [67], and Rey’s figure test [69], among others. These tests evaluated five cognitive domains: learning and memory, attention and executive function, constructive ability, praxis, and language (see Table 2) [70,71,72]. Our frame of reference for this selection was the Buschke & Fuld memory test, the Visual Reproduction Test (VRT), certain verbal and phonetic fluency tests, associated pair learning tests, and the Benton Test. Apart from the references cited, the selection was also made by taking into account the time of application, the existence of standard scales, and the use of the tests within our context. Subsequently, we reviewed and extended the protocol to include an assessment of working memory through the Inverse Digits Test and the Barcelona Test, as well as the Direct Digits Test to assess attention and another test to assess language in its lexical aspect or modality: a naming test. Obviously, the sensitivity of the assessment increases if there are a large number of tests, aiding the classification of MCI into different subtypes. However, it may also be problematic evaluating a cognitive domain in a redundant manner with too many tests.

In order to reduce the time required to apply the battery of tests, which was considered to be too long, a factor analysis and a main components analysis were performed to see how the tests being applied were conceptually grouped and which had more weight in the assessment. This exploratory factor analysis highlighted two predominant cognitive dimensions: memory tests on the one hand; and executive functions and praxis on the other hand [73]. In relation to the determination of the cut-off points, a deterioration cut-off point of −1.5 SD was established in each of the tests, coinciding with most of the literature reviewed and with more conventional criteria [6,70,74,75]. The results based on these psychometric criteria allowed a sample of 140 individuals to be classified into three MCI subtypes, yielding the following percentages: healthy individuals (46.71%), aMCI (6.42%), mMCI (22.14%), and naMCI (25.71%).

## 3. Problems Related to the Conceptualization and Operationalization of Maintaining the Functionality of Activities of Daily Living

Although maintaining the functionality of activities of daily living (ADL) is an essential neuropsychological criterion in the detection, diagnosis, and differentiation of MCI with respect to dementia [5,29], it is not exempt from problems in terms of its conception, assessment, and the thresholds of deterioration, as well as in terms of the relationship between cognitive and functional impairment. Some of the issues that arise remain unresolved, including: what are functional or daily life activities? Do these abilities refer to instrumental activities or to some basic activities as well? Is it necessary to distinguish them according to subject variables, for example, socio-educational level or age? And, are they involved equally in different MCI subtypes?

Firstly, it is unclear whether a consensus exists when referring to functional skills and to what extent this concept has been modified, particularly as one of the essential criteria to differentiate MCI from dementias was proposed to be that the individual should preserve independence in functional abilities [2]. Currently, there are subtle deficiencies in instrumental ADL (IADL) in MCI [3], as individuals initially present a deficit in activities like money management, transportation use, medication management, etc., all of which require a higher cognitive level than more basic activities such as personal grooming [76,77]. ADLs are usually evaluated through interviews and the scales applied to the person with MCI and their next of kin are considered to be reliable tools. However, as recently suggested [12], it may be useful to also include tests where the individual him/herself performs ADLs of different levels of difficulty in order to establish more precise and operative criteria. As occurs with cognitive assessment, it is necessary to reach a consensus on which scales, tests, and questionnaires are sufficiently sensitive to detect alterations in functional activity, and whether this information should be collected from both the individual and his or her immediate environment. Likewise, the threshold or point at which the functionality of ADL is to be considered as deteriorated in the individual with MCI must be established [24].

As MCI evolves, both cognitive and functional abilities will deteriorate in individuals who develop dementia, and individuals with MCI who have impaired IADL are at higher risk of progressing to that state [78]. The decrease in functional ability is associated with a decrease in the overall cognitive functioning of the individual with MCI [8]. This raises the question of whether this deficit occurs in all MCI subtypes and whether it happens to the same magnitude, which would make functional activity useful in the diagnosis of MCI and its classification into subtypes. In this respect, the relationship between the pattern of cognitive performance in neuropsychological tests and subtle deficits in IADL was recently analyzed in a group of aMCI and naMCI subjects [24]. In addition, this study incorporated a concept that will surely be analyzed in the forthcoming years: the degree of awareness of the individual’s MCI deficit and its ability to predict the functional impairment of MCI subtypes. The results showed that both executive functions and memory, together with the lack of deficit awareness, predicted the early deterioration of the IADL in the MCI subtypes. This led to an estimation of the patient’s prognosis, as well as to the design of individualized interventions [24].

An exhaustive review recently showed the existence of a large variety of instruments to evaluate IADL in subjects with MCI [76], although originally most of these instruments were designed for patients suffering from dementia. While informant-report rating questionnaires are more frequently used than performance-based tests, the latter highlighted differences in IADL functioning between subjects with MCI, patients with dementia, and cognitively healthy persons. Likewise, these tests show which activities are consistently impaired in subjects with MCI, such us financial management capacity [79]. In terms of the different MCI subtypes, deficits in IADL were more evident in aMCI than in naMCI, and in multiple-domain MCI than in single-domain [76]. These findings are relevant to determine the subtype of MCI, confirming that alterations in IADL are not homogeneous in the different MCI subtypes. Therefore, we consider that an appropriate evaluation of MCI should not only include a neuropsychological assessment, but also other factors related to IADL like financial management capacity, preparing a balanced meal and the correct self-administration of medication [80].

## 4. Analysis of Some of the Methods to Diagnose MCI and Its Possible Sub-Types

As different cut-off points can be adopted to establish impairment thresholds that identify deficits in the performance of specific cognitive tests, metric criteria can be imprecise when identifying MCI subjects, and even more when classifying them into subtypes. Several criteria have been frequently applied when using SD as a reference measure: 1.0 SD, 1.5 SD, or even 2.0 SD. In the case of a perfectly normal distribution, the probability that the score in a test is between 1.0 SD and −1.0 SD of the mean is 68.26%. Hence, the probability that the score obtained falls outside that area is 31.74% and when this range of exclusion is distributed between the two tails of a normal distribution, it can be said that 15.87% of the scores in that test would be −1.0 SD or more away from the mean. Therefore, as there is a very high probability of a score falling into the exclusion zone, this criterion of −1.0 SD from the mean would augment the number of cases with MCI and therefore increase the number of false positives (individuals diagnosed with MCI when they are, in fact, cognitively healthy). This would suggest that this cut-off has low specificity.

Alternatively, if 1.5 SD is used as the failure threshold, then 86.64% of the scores would be included in the range of ±1.5 SD from the mean when the distribution is perfectly normal. This would exclude 13.36% of the total scores or 6.68% in each of the distribution tails. This criterion is less demanding than the previous one, which could lead to a lower sensitivity in identifying MCI cases, thereby increasing the probability of false negatives (individuals with MCI who are identified as healthy). In the less frequent case of using 2.0 SD as the deterioration threshold, 95.44% of the scores in a test would be included in the area of ±2.0 SD in normal distributions, which would leave only 4.56% below the threshold. Following the same logic as that applied above, only 2.28% of the scores would be −2.0 SD or more away from the mean and thus, this criterion can be considered as less sensitive to identify cases of MCI as it only detects really low scores that are much more frequent in dementias than in MCI.

In general, any diagnostic criteria should be capable of detecting all cases, not only the severe cases, but also the moderate or mild cases. However, this can pose a problem with MCI because if the performance in a particular cognitive test is considered as a continuum, the criterion that unequivocally detects the existence of a disorder (such as using 2.0 SD) probably identifies a case of dementia and not MCI. Therefore, the focus should be placed on what might be considered as mild cases, for which the choice of a cut-off point is not simple either. In this sense, it has been concluded that “*more strict cut-points on cognitive tests of −1.5 or −2 SDs below normative means generally trade modest gains in specificity for larger losses in sensitivity, and several studies suggest that a cut-point for impairment of −1 SD below normative means provides an optimal balance of sensitivity and specificity*” [9,52,58,81].

On the other hand, it is important to note that the distribution of the scores has to be normal in order to be able to use the SD as a reference measure. This is frequently not the case when working with older populations, as the variability of this population’s scores increases considerably and their scores are often not normally distributed. In these cases, it is more appropriate to use other metric criteria, such as standardizing scores or percentiles, such as those being used at the Mayo Clinic (see in the next paragraph).

In addition to being able to identify a specific and sensitive cut-off point to detect alterations in the performance of neuropsychological tests that diagnose MCI, it is also of the utmost importance to have scales or standardized data specific for older adults that, in addition to their age, take into account their educational level and the population to which they belong. The number of years of schooling is a fundamental moderating variable in the final outcome of most neuropsychological tests [82,83], thus it is essential to consider it when elaborating standardized data. Depending on the level of education of the individual, the same score in a test may or may not indicate a deficit. Such differentiation is not possible if the cut-off (or SD) alone is taken into account. Researchers at the Mayo Clinic have dedicated more than a decade to a program aimed at providing specific standardized data for the neuropsychological assessment of older adults: Mayo’s Older Americans Normative Studies—MOANS [84,85,86,87] and Mayo’s Older African American Normative Studies -MOAANS [88]. The normative procedures used in these two projects are: (a) the use of mid-point age intervals to maximize the information available at each age [89]; (b) the conversion of each raw score to a percentile rank, such that each test’s raw score distribution at each mid-point age can be “normalized” by assigning standard scores (i.e.,: mean = 10; SD = 3) based on the actual percentile ranks (see [81] for a more extended explanation); and (c), linear regressions are applied to the normalized age corrected MOANS scaled scores for each test to further adjust for education. In this way, the standardized scores are based on percentiles and they avoid the aforementioned problems associated with normal distributions. In Spain, the Spanish Multicenter Normative Studies (NEURONORMA Project) is replicating the standardization on a Spanish population through a similar procedure to that used at the Mayo Clinic but with different neuropsychological tests [90]. Currently, we are also working on the creation of standardized scales for some of the tests that make up our assessment battery. For example, we are working towards the standardization of the Spanish version of the California Verbal Learning Test (CVLT) for use with older adults, known as TAVEC (Test de Aprendizaje Verbal España-Complutense—Spain-Complutense Verbal Learning Test) [66], taking into account the number of years of schooling as performed in the MOANS and NEURONORMA projects. This is being done as there are no recent standardized data for this population that also take into account the variance that the number of years of schooling may be introducing in the results of this test.

In addition to the more psychometric perspective discussed above, there is another set of statistical methods that cannot be overlooked and that can be used to identify possible performance profiles, that is, to classify individuals with MCI and distinguish them from healthy individuals. Within these methods, cluster analysis should be highlighted as it is considered a multivariate statistical technique focused on the classification of variables in function of the similarity between them. The advantages of this type of analysis are that it is less restrictive and more flexible in its assumptions, it does not require linearity or normality, and it also allows for categorical and not only quantitative variables. Nevertheless, an important problem of this methodology is that, a priori, the groups or clusters are unknown and it is the researcher who must determine the optimum number of clusters that the population can be divided into [91]. In the case of MCI, this would pose the following problems: (a) how many subtypes should be considered, two (amnesic and non-amnesic), three (aMCI, naMCI, and mMCI), or even four or more subgroups; and (b), the discriminability or relevance of the tests applied is fundamental in this type of method, as there may be tests that are more sensitive than others when detecting possible alterations, affecting the clusters obtained. Both these problems require prior decision-making. When this type of statistical analysis was used to analyze the groups aMCI, naMCI, and mMCI, no clear limits were found between them (well-defined cognitive profiles of deterioration were not identified), in contrast to when criteria were considered in a more purely psychometric manner (e.g., SD) [70]. Nevertheless, cluster analyses have been used to determine different patterns of episodic memory impairment in adults with memory complaints [92].

Due to the limitations of cluster analysis noted, latent class analysis (LCA) has been assessed as an alternative classification method, as it provides less arbitrary criteria to determine the number of groups in the population [93]. Specifically, it is based on the idea that an unobserved (latent), discrete (categorical) variable describes the relationships between the variables manifested. In the specific case of MCI, this technique would allow us to identify the relationships between the scores in the different neuropsychological tests applied, assuming that the structure of the underlying relationships between them is explained by a categorical, exhaustive, and mutually exclusive latent variable like the subtype of MCI. As such, a LCA would try to identify each of the MCI subtypes through the scores obtained in the neuropsychological tests. Indeed, it is noteworthy that three subtypes of MCI were obtained when using an LCA (aMCI, naMCI, and mMCI) [94], corroborating the existence of three types of profiles with cognitive impairment among older participants not identified as being healthy.

The importance of the evolution of the disorder on the diagnosis of MCI cannot be overlooked, as it is well-known that individuals may revert to normal, stabilize, or develop dementia. In this sense, the importance of having specific and sensitive diagnostic criteria in order to reduce the number of false positive MCI diagnoses has been highlighted [38]. Therefore, a longitudinal assessment is fundamental for this pathology and Latent Transition Analysis (LTA) is a useful method to identify possible changes between groups over time. One example of this approach focused on using Markov transition models to study diagnostic changes at different intervals between the baseline and follow-up assessments [95]. Accordingly, it was considered that “*Longitudinal follow-up will determine whether varying diagnostic criteria improves sensitivity and specificity of the MCI diagnosis as a predictor for dementia*” [65].

Finally, despite taking into account the possible evolution of MCI by carrying out longitudinal studies, it is known that interindividual variability is dissipated by working with the average scores of the group, leading to a loss of precision in the diagnosis. Therefore, if an increase in the ability to detect changes in the cognitive or functional performance of an individual is needed to accurately and promptly diagnose MCI, it would be convenient to analyze the evolution that an individual shows in the performance of tests over time, instead of focusing only on the average scores of the group obtained on the basis of standardized criteria. For this reason, our tendency would be to apply *hierarchical linear models* to study individual change in cognitive function.

## 5. Social Factors Associated to Mild Cognitive Impairment

As humans, we are social beings and we have an inherent need to belong to a social network [96]. It is well known that social support is critical for physical and psychological health [97], and that social isolation, an objective measure of poor social integration, is related to increased morbidity and mortality [98]. Social support not only involves network size and the frequency of social interactions, but also the emotions of the person. Despite having a social network, a person may feel that he/she is not satisfied with his/her social interactions and experience emotional isolation, presenting feelings of loneliness [99]. Although social isolation and loneliness can occur at any time in life, it is more prevalent in older adults [100].

There is now a growing interest in studying the contribution of social support to cognitive function in older adults, e.g. [101,102,103]. Although some studies failed to identify an association between social networks and cognitive function [104,105], there is now ample evidence indicating that older individuals that maintain a good social support network and that participate more in social activities show enhanced cognitive abilities and experience less cognitive deficits over time [106,107,108]. By contrast, negative social interactions, including unsympathetic behavior and rejection, are associated with an increased incidence of naMCI [109]. Interestingly, the negative association between a lack of social support and cognition in older people is stronger in women than in men [110].

Several studies have indicated that social isolation is a risk factor for cognitive decline in aging [110,111]. In older individuals, loneliness is inversely related to different cognitive measures independent of depression, including episodic, semantic and working memory, processing speed, and visuospatial ability [112,113,114]. In addition, several prospective cohort studies have analyzed whether loneliness is associated with age-related cognitive decline. Thus, in a 7.5-year longitudinal study, greater emotional support predicted better cognitive function in older adults after controlling for baseline cognitive function and known sociodemographic, behavioral, psychological, and health status predictors of cognitive aging [106]. Subsequently, loneliness was shown to predict cognitive decline over four to 12 years, although there was some discrepancy about the cognitive domains affected [112,115,116,117]. Thus, while there are reports that loneliness is associated with a faster decline in processing speed and delayed visual memory independently of depression and social contacts [112,115], loneliness has also been proposed as a factor that predicts the incidence of aMCI (according to Petersen’s criteria) [117]. Recently, it was observed that individuals considered at risk of MCI and dementia had smaller network sizes and were involved in less community activities than individuals that had normal cognitive function [118].

Prospective studies have also identified that leisure activity and sociodemographic factors are relevant to MCI onset [119,120]. Thus, less participation in leisure activities (e.g., reading, writing, crossword puzzles, board or card games, group discussions, or playing music) is related to an increased risk for aMCI in older individuals [121], while participation in leisure, social, and cognitive activities increases cognitive reserves and delays the manifestation of MCI symptoms [122]. Moreover, older adults with MCI participate less in leisure activities like reading, attending a senior university, or using a computer, and show less physical, intellectual, and social interaction activities compared to subjects with normal cognition [123]. Interestingly, activities stimulating cognition not only slow down cognitive decline in normal adults, but also in patients with dementia [124,125]. In a recent randomized controlled trial on older adults with MCI, a long-term leisure activity program that included dancing and playing a musical instrument improved memory and general cognition when compared to a health activity program [125]. Disruptions in social activities and feelings of loneliness are behavioral symptoms that result from subtle neuropathological changes that may occur before MCI and dementia commences. In fact, both impaired social cognition and diminished sociocognitive skills are evident in prodromal dementia [126,127].

In summary, social isolation, loneliness, and less participation in leisure activities seem to be important risk factors associated with the progression from normal cognition to MCI. The controversy around the impact of these social factors in cognition may be due to the fact that some studies use small samples or cross-sectional designs rather than longitudinal studies of large numbers of subjects. Future studies should take into account these and other socio-demographic factors in order to better understand their role in the appearance of certain MCI subtypes. In addition, it would be interesting to determine whether the existence of feelings of loneliness in association with aging may be a useful diagnostic criterion to identify “non-specific” MCI and/or other sub-types of MCI. Effective interventions that promote access to social resources, social inclusion, and that support an active social lifestyle in older adults represent a plausible strategy to not only reduce the risk for MCI, but also to promote human and societal well-being. Undoubtedly, effective interventions for older adults diagnosed with MCI are crucial issues to be addressed in aging care [80].

## 6. Conclusions

As we have seen throughout this review, MCI began to be considered as distinct sub-types in the 1990s, first as two sub-types, aMCI and naMCI, and then as three, by adding mMCI. Subsequently, four subtypes were defined: single and multi-domain aMCI and naMCI [7]. However, since then, additional MCI sub-types have not been identified and unfortunately, a consensus among experts regarding the number and types of MCI has still to be reached. In fact, the entity itself is still considered very heterogeneous and the DSM-5 criteria have not clarified the situation [128]. As a result, only four subprofiles are currently considered, and in the last decade, there has apparently been little or no evolution in this sense. It is unclear whether it makes sense to continue to refer to a global and generic MCI today. We do not think this to be the case and that is why, in this paper, we have proposed addressing this troublesome topic, analyzing the difficulties found in the attempts to describe the subgroups or subtypes of MCI of every kind—conceptual, methodological, and operational—, an approach that it is still not very common. In fact, even at present, some international consensus groups fail to even mention the existence of subtypes of MCI [25,26]. Nevertheless, the need and importance of addressing the possible subtypes or subgroups of MCI has been expressed [129], and of even establishing criteria defining the possible MCI subtypes based on reversion or non-reversion to normality [130].

In conjunction with a review of the current literature, we present some of the difficulties found during our own research in this area. In an attempt to resolve these, we have proposed both conceptual and operational guidelines that will help to clarify the issue of categorizing and classifying MCI. This no doubt requires paying greater attention to variables that had not previously been taken into account, or not systematically, as well as the introduction of other variables related to the diagnosis of MCI (see also [30]). As such, we highlight the importance of some of the currently relevant and psychosocial factors: social isolation and loneliness. In addition, and in line with others [131,132,133], we also stress how a series of personal variables (educational level and cultural traits, factors related to personality, cognitive reserve) and some co-morbidities (even of a neuropsychiatric nature), could interact with and be associated with certain manifestations of MCI.

Among the unresolved problems associated with MCI are that of the stability versus instability of the syndrome, the lack of knowledge about certain etiologies and risk factors, or the successive stages of a single process. In terms of mNCD, for which no distinct subtypes are defined, cognitive deficits may be evident at the very early stages of certain neurodegenerative diseases (associated with LB or frontotemporal dementia, AD, etc.) or due to other etiologies (AIDS, craneoencephalic trauma, etc.). There are currently well-established biomarkers of several neurodegenerative diseases that help define whether MCI is due to an underlying neurodegenerative disease. Nevertheless, future studies will be necessary to find new biomarkers that identify the etiology of other undifferentiated and less specific cognitive impairment disorders, helping us to define the construct both conceptually and operationally. With regard to these potentially undifferentiated types of cognitive impairment, Subjective Cognitive Impairment (SCI) should perhaps be mentioned, a construct that has replaced Subjective Memory Impairment (SMI). SCI might represent an early sign preceding dementia, yet it is still used to achieve unequivocal status in relation to MCI.

The discovery of blood-based biomarkers that might predict progression from MCI to a cognitive disease has become an important focus of neurobiology research. For instance, plasma beta secretase 1 (BACE1) activity was recently shown to increase significantly in MCI that converted to probable AD dementia after three years relative to patients with stable MCI [134]. This measurement showed a high sensitivity (85%) and specificity (88%), indicating that plasma BACE1 activity could be a useful biomarker to identify progression from MCI to AD. Yet if defining new biomarkers is necessary to identify distinguishing criteria, better understanding the implication of affective symptoms—and not only cognitive ones—is also crucial. Indeed, while classifying individuals with MCI into different subtypes has relied almost exclusively on the number and type of cognitive processes or functions affected, the current trend is to also introduce affective aspects and not just cognitive ones. This has led to the analysis and study of negative affective symptoms, such as apathy, sadness, and psychomotor slowness and passivity, further complicating this field [128]. A new construct was recently described, Mild Behavioral Impairment (MBI), in which non-cognitive and strictly behavioral aspects are considered in the framework of cognitive impairment in the elderly. Although MBI was not addressed in this review as it cannot be currently related to the different subtypes of MCI, we should bear in mind that cognitive and emotional aspects are interrelated. Hence, both aspects should probably be integrated when adopting conceptual and methodological approaches to MCI.

Finally, it is absolutely necessary to reach a consensus about the tests, norms, scales, etc. to be applied in the cognitive, functional, and affective evaluation of MCI, and regarding the thresholds and cut-off points for deterioration, the selection of samples and the clinical criteria. Until such agreement is reached, the very definition of MCI, its possible subtypes, prevalence, and incidence, as well as the diagnosis of MCI, will continue to be problematic.

In conclusion, we believe that the attempts to categorize people with MCI on the basis of different criteria are not in vain. Indeed, as we learn more about the evolution of the cognitive system in older adults, we see that there are countless factors or variables that may affect its integrity or deterioration. Therefore, an increasingly more precise and specific understanding of the risk factors, biomarkers, co-morbidities, psychosocial factors, personality traits, etc. may help to systematize this field, which is currently still far from this goal.

## Figures and Tables

**Table 1:** Sub-classification of the different MCI subtypes.

References	Cognitive Processes Evaluated	MCI Subtypes
Petersen [2]	Episodic memory	aMCI
Petersen et al. [5]	Episodic memory	aMCI
	Language	Single non-memory domain MCI
	Executive function	Multiple domains, slight impairment MCI
	Visuospatial skills	
Petersen [6]	Episodic memory	Single domain aMCI
Petersen & Negash [7]	Language	Multiple domain aMCI
	Executive function	Single domain naMCI
	Visuospatial skills	Multiple domain naMCI
Edmonds et al. [14]	Memory	aMCI
	Language	Dysnomic MCI
	Attention	Dysexecutive MCI)
	Executive function	Cluster-Derived Normal(within normal limits on cognitive testing)
Eppig et al. [15]	Episodic memory	aMCI
Libon et al. [16]	Language	dMCI (executive and processing speed deficits)
	Executive functioning	mx MCI (memory and language deficits)
	Processing speed	
	Visuo-construction	
Rosenberg et al. [17]	Episodic memory recall	aMCI
	Language	exMCI
	Attention	Both aMCI and exMCI
	Executive function	Neither aMCI nor exMCI
	Visuospatial function	
Mansbach et al. [18]	Verbal memory	aMCI
	Executive control functions	Executive MCI
	Attentional capacity	Multi-domain MCI
		Undifferentiated MCI
Albert et al. [20]	Episodic verbal memory	aMCI
Saunders & Summers [22]	Language	
Putcha & Tremont [24]	Executive function	
	Attention	naMCI
	Working memory	

Note: aMCI (amnestic MCI); naMCI (non-amnestic MCI); dMCI (dysexecutive MCI); mxMCI (mixed or multi-domain MCI); exMCI (executive dysfunction-MCI); non-exMCI (no executive dysfunction-MCI).

**Table 2:** Cognitive domains and tests.

Cognitive Domains	Tests
Episodic memory and learning	Verbal Learning Test Spain Complutense (*Test de Aprendizaje Verbal España Complutense—TAVEC*) [66]
	The Rey-Osterrieth complex figure [69]
Working Memory	Inverse Digits, Barcelona Test [67]
Language	Semantic and phonemic fluency, Barcelona Test [67]
Attention and Executive function	Direct Digits, Test Barcelona [67]
	Comprehensive Trail-Making Test (CTMT), A and B [68]
	Alternating graphs and loops, Barcelona Test [67]
Constructive praxis	The Rey-Osterrieth complex figure [69]
	Praxis constructive graphics, Barcelona Test [67]
Ideomotor praxis	Mimicking the use of objects and Symbolic gestures of communication, Barcelona Test [67]
